# Group II Metabotropic Glutamate Receptors as Targets for Novel Antipsychotic Drugs

**DOI:** 10.3389/fphar.2016.00130

**Published:** 2016-05-20

**Authors:** Carolina Muguruza, J. Javier Meana, Luis F. Callado

**Affiliations:** ^1^Department of Pharmacology, University of the Basque Country, UPV/EHULeioa, Spain; ^2^Centro de Investigación Biomédica en Red de Salud MentalMadrid, Spain

**Keywords:** antipsychotic, glutamate, human brain, mGlu_2_R receptors, schizophrenia

## Abstract

Schizophrenia is a chronic psychiatric disorder which substantially impairs patients’ quality of life. Despite the extensive research in this field, the pathophysiology and etiology of schizophrenia remain unknown. Different neurotransmitter systems and functional networks have been found to be affected in the brain of patients with schizophrenia. In this context, postmortem brain studies as well as genetic assays have suggested alterations in Group II metabotropic glutamate receptors (mGluRs) in schizophrenia. Despite many years of drug research, several needs in the treatment of schizophrenia have not been addressed sufficiently. In fact, only 5–10% of patients with schizophrenia successfully achieve a full recovery after treatment. In recent years mGluRs have turned up as novel targets for the design of new antipsychotic medications for schizophrenia. Concretely, Group II mGluRs are of particular interest due to their regulatory role in neurotransmission modulating glutamatergic activity in brain synapses. Preclinical studies have demonstrated that orthosteric Group II mGluR agonists exhibit antipsychotic-like properties in animal models of schizophrenia. However, when these compounds have been tested in human clinical studies with schizophrenic patients results have been inconclusive. Nevertheless, it has been recently suggested that this apparent lack of efficacy in schizophrenic patients may be related to previous exposure to atypical antipsychotics. Moreover, the role of the functional heterocomplex formed by 5-HT_2A_ and mGlu_2_ receptors in the clinical response to Group II mGluR agonists is currently under study.

## Introduction

Schizophrenia is a severe, chronic, and disabling mental disorder affecting approximately 0.6% of the population worldwide (McGrath et al., 2008). Among psychiatric disorders, it is considered the most disabling one, requiring a disproportionate share of mental health services (Mueser and McGurk, 2004). Individuals diagnosed with schizophrenia have impaired social and occupational functioning. Thus, schizophrenia is placed among the world’s top leading causes of years lived with disability (World Health Organization, 2008; Vos et al., 2015), being also the seventh most costly medical illness in our society (Freedman, 2003).

The clinical features of schizophrenia are clustered in three categories: positive symptoms, negative symptoms and cognitive deficits. Positive or psychotic symptoms include delusions (false beliefs held with strong conviction in spite of contradictory evidence), hallucinations (perceptions in the absence of external stimulus, commonly experienced as hearing voices distinct from one’s own thoughts), thought disorder (e.g., loose associations), and abnormal psychomotor activity (e.g., grossly disorganized behavior, posturing, or catatonia). Negative symptoms comprise social withdrawal, impairments in initiative and motivation, a reduced capacity to recognize and express emotional states and poverty in the amount or content of speech. Cognitive impairments include disturbances in selective attention, working memory, executive control, episodic memory, language comprehension, and social-emotional processing. Symptomatic onset occurs in late adolescence and early adulthood in males and somewhat later in females, who tend to be less severely affected (Abel et al., 2010). The course of schizophrenia is typically characterized by psychotic exacerbations or relapses alternating with periods of partial remissions.

The principal pharmacological treatment for schizophrenia is antipsychotic medication. In general terms, antipsychotic drugs are effective in reducing the severity of positive symptoms such as hallucinations and delusions and have made it possible for many individuals with schizophrenia to live outside hospital settings. Nevertheless, antipsychotics have minimal impact on both negative symptoms and cognitive impairments (see Miyamoto et al., 2012 for review). Thus, the treatment of schizophrenia with antipsychotics rarely, if ever, produces a cure or entirely reverses symptoms of the illness. Only 5–10% of persons with schizophrenia successfully achieve a full recovery with or without these medications. There is a good response to antipsychotic medication in 30–40% of patients. However, about 20% are resistant to standard antipsychotics and an additional 30–40% show an improvement but are residually symptomatic despite antipsychotic treatment (Smith et al., 2009).

## The Glutamate Hypothesis of Schizophrenia

Glutamate is the major excitatory neurotransmitter in the brain. It interacts with two types of receptors: (i) the ionotropic receptors, with NMDA, Kainate, and AMPA receptor subtypes connected to or representing ion channels, and (ii) the metabotropic glutamate receptors (mGluRs), which activate G protein-coupled signal transduction and comprising groups I to III with a total of eight identified subtypes (Nakanishi, 1992).

The observation that the administration of phencyclidine (PCP) and the dissociative anesthetic ketamine —two NMDA receptor antagonists— could mimic schizophrenia symptoms in healthy individuals led to the hypothesis of a functional impairment of NMDA receptors in this disease (Javitt and Zukin, 1991; Olney and Farber, 1995; Stone et al., 2008). Importantly, it was described that NMDA receptor antagonists, besides inducing positive-like symptoms, also found to be induced by other stimulant drugs such as amphetamine and other dopaminergic agonists, were also able to evoke cognitive- and negative-like symptoms (Krystal et al., 2005). Since dopamine release is under control of NMDA receptors in several brain circuits, some authors sustain that the glutamatergic dysfunction may be underlying the dopaminergic deficits found in schizophrenia (Javitt, 2010).

Additionally, genome-wide association studies have shown that genes involved in glutamatergic neurotransmission and synaptic plasticity, e.g., mGluR3 (*GRM3*), glutamate ionotropic receptor NMDA type subunit 2A (*GRIN2A*), serine racemase (*SRR*), glutamate ionotropic receptor AMPA type subunit 1 (*GRIA1*) or neurogranin (*NRGN*), are associated with schizophrenia (Stefansson et al., 2009; Schizophrenia Working Group of the Psychiatric Genomics Consortium, 2014). Moreover, postmortem and neuroimaging studies have found that several components of glutamatergic signaling system are affected in schizophrenic patients (Gao et al., 2000; Stone, 2009; Coyle et al., 2012). Thus, drugs targeted to restore the glutamatergic imbalance could provide better outcomes than those obtained with current antipsychotics in the treatment of schizophrenia. In this sense, preclinical and clinical studies have provided evidence for mGluRs 2, 3, and 5, muscarinic receptors M1 and M4 or the Glycine transporter GlyT1 among others as potential targets to retrieve the glutamatergic functioning in schizophrenia (Field et al., 2011).

## Group II Metabotropic Glutamate Receptors

**FIGURE 1:**
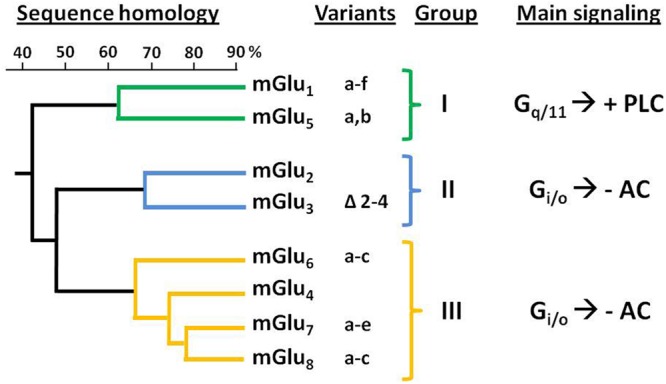
**Classification and sequence homology dendrogram of mGluRs.** The known splice variants and main signaling pathways are indicated. –AC, inhibition of adenylyl cyclase activity; +PLC, activation of phospholipase C.

Genes encoding eight mGluR subtypes —many of them with multiple splice variants— have been identified and are classified into three groups (I–III) according to their sequence homology, coupling mechanism and pharmacology (Conn and Pin, 1997; Niswender and Conn, 2010; **Figure 1**). Group II includes mGlu_2_ and mGlu_3_ receptors, that are coupled predominantly to G_i/o_ proteins, which mediate the downstream inhibition of adenylyl cyclase activity, modulation of voltage-dependent ion channels (inhibition of calcium and activation of potassium channels), and the regulation of other downstream signaling partners via released Gβγ subunits. Recent studies have shown that Group II mGluRs can also modulate additional signaling pathways, such as activation of PI3K and MAPK pathways (Iacovelli et al., 2002; Niswender and Conn, 2010; Nicoletti et al., 2011).

The mGlu_2_ and mGlu_3_ receptors share about 70% of their amino acid sequence (Pin and Duvoisin, 1995). The gene encoding the human mGlu_2_R (*GRM2*) has been mapped to chromosome 3p21.1-p21.2 (Marti et al., 2002a). For its part, mGlu_3_R gene (*GRM3*) was mapped to human chromosome 7q21.1-q21.2 (Scherer et al., 1996). While no splicing variants have been reported for *GRM2*, alternative splicing has been described for *GRM3* leading to four different variants: full length mGlu_3_R, *GRM3*Δ*2* (lacking exon 2), *GRM3*Δ*4* (lacking exon 4), and *GRM3A2*Δ*3* (lacking exons 2 and 3); being the *GRM3*Δ*4* the most abundant one (Sartorius et al., 2006). Despite this variant lacks the transmembrane domain—which is encoded by exon 4— it has been shown that it can be translated in cells, thus suggesting its potential function as a unique glutamate receptor (Niswender and Conn, 2010).

Group II mGluRs are widely expressed throughout the central nervous system. The expression levels are moderate to high in different brain regions such as the prefrontal cortex (PFC), the dorsal and ventral striatum, the thalamus, the hippocampus, and the amygdala (Petralia et al., 1996; Wright et al., 2001; Gu et al., 2008); regions that have been shown to be involved in cognition and emotional states. The mGlu_3_Rs expression across these regions is more disperse than that of mGlu_2_Rs (Ohishi et al., 1993; Gu et al., 2008). In the PFC mGlu_2_Rs present a high but restricted expression with a bilaminar distribution in layer I and layer Va while mGlu_3_Rs distribution is more homogenous throughout the cortex with a slight higher expression in layers I–III than in layers IV–VI (Marek, 2010). At the neuronal level, mGlu_2_Rs are localized at the perisynapse (Cartmell and Schoepp, 2000) mainly acting as autoreceptors where they function as a feedback negative mechanism to suppress the excessive glutamate release keeping the homeostasis of the synapse (Cartmell and Schoepp, 2000; Schoepp, 2001). However, both presynaptic and postsynaptic cortical immunoreactivity of mGlu_2_Rs has been described. For its part mGlu_3_Rs immunoreactivity has been shown mainly presynaptic (Neki et al., 1996; Ohishi et al., 1998; Tamaru et al., 2001) and whereas mGlu_2_Rs expression is restricted to neurons, mGlu_3_Rs are also found on glial cells (Ohishi et al., 1993; Tamaru et al., 2001) where they may interact with glutamate transporters (Aronica et al., 2003).

Besides modulation of glutamate physiology, Group II mGluRs, also control the neurotransmitter release of other systems acting as heteroreceptors in GABAergic, dopaminergic, noradrenergic, or serotonergic synapses (Cartmell and Schoepp, 2000).

A limited number of molecules possess agonist activity across all mGluRs. The endogenous agonist L-glutamate, L-CCG-I [(2S,10 S,20 S)-2-(carboxycyclopropyl)glycine] and ABHxD-I (2-aminobicyclo[2.1.1]hexane-2,5-dicarboxylic acid-I) are the most potent (Acher, 2011). More recently, systemically active and highly selective agonists of Group II mGluRs have been developed, providing valuable insights into the *in vitro* and *in vivo* functions of these receptors (Niswender and Conn, 2010). LY354740 ((1S,2S,5R,6S)-2-Aminobicyclo[3.1.0]hexane-2, 6-di carboxylic acid) was the first Group II mGluR selective agonist reported to exhibit a nanomolar affinity (Monn et al., 1997). It has been followed by more recent compounds, including LY379268 ((1S,2R,5R,6R)-2-amino-4-oxabicyclo[3.1.0]hexane-2,6-dicarboxylic acid), now a commonly used tool for studies of Group II mGluR function (Schoepp et al., 1999). These compounds are highly selective for Group II mGluRs relative to other mGluR subtypes but do not differentiate between mGlu_2_R and mGlu_3_R. Other Group II selective agonists have been described with submicromolar affinity, including (2R,4R)-APDC ((2R,4R)-4-aminopyrrolidine-2,4-dicarboxylate) and DCG-IV ((2S,2′R,3′R)-2-(2′,3′-dicarboxycyclopropyl)glycine). Additiona lly, an analog of LY354740 with a methyl substituent at the C4α-position was reported to have mGlu_2_R agonist and mGlu_3_R antagonist activity (Dominguez et al., 2005). Thus far, no orthosteric antagonists have been discovered that are entirely specific for Group II mGluRs. However, LY341495 ((2S)-2-amino-2-[(1S,2S)-2-carboxycycloprop-1-yl]-3-(xanth-9-yl) propanoic acid) provides relatively high selectivity with nanomolar potency as a Group II mGluR antagonist with submicromolar to micromolar potencies at all other mGluR subtypes (Schoepp et al., 1999).

To date, besides orthosteric ligands, multiple selective positive allosteric modulators (PAMs) of mGlu_2_R have been identified. The majority are structurally related to either LY487379 (2,2,2-trifluoro-N-[4-(2-methoxyphenoxy)phenyl]-N-(3-pyridinylmethyl)ethanesulfonamide hydrochloride) or BINA (biphenyl-indanone A), two prototypical mGlu_2_R PAMs (Conn et al., 2009; Niswender and Conn, 2010). Many of these compounds are highly selective for mGlu_2_R and do not potentiate responses to activation of mGlu_3_R or any other mGluR subtype (Cid et al., 2015). In addition, group II mGluR negative allosteric modulators (NAMs) have also been developed, but in this case acting at both mGlu_2_R and mGlu_3_R (Hemstapat et al., 2007; Woltering et al., 2008a,b).

## Alterations of Group II mGluRs in Schizophrenia

### Postmortem Brain Studies

Different approaches have been used to determine the possible alterations of both, mRNA and protein expression, of Group II mGluRs in the postmortem brain of schizophrenic subjects. The majority of the findings suggest that the level of expression of *GRM3* mRNA is unaffected in schizophrenia (Ohnuma et al., 1998; Richardson-Burns et al., 2000; Egan et al., 2004; Bullock et al., 2008; Ghose et al., 2008; Gonzalez-Maeso et al., 2008). Fewer studies have investigated *GRM2* mRNA expression in postmortem human brain of schizophrenic subjects. Semi-quantitative approaches such as *in situ* hybridization have reported unaffected levels of *GRM2* mRNA in thalamus (Richardson-Burns et al., 2000), and higher *GRM2* mRNA expression in the PFC white matter (Ghose et al., 2008). However, quantitative real-time PCR assays showed lower level of expression of *GRM2* mRNA in the PFC (Gonzalez-Maeso et al., 2008) and cerebellum (Bullock et al., 2008) of schizophrenic subjects.

Studies using immunolabeling techniques have reported different outcomes in regard to Group II mGluRs protein expression levels in schizophrenia. Differentiation of mGlu_3_R from mGlu_2_R has been problematic because of the lack of selective ligands and antibodies. An early study, using non-specific antibodies that detect both mGlu_2_ and mGlu_3_ receptor proteins, found no change in mGlu_2/3_R expression in the PFC (BA46) of schizophrenic subjects compared to controls (Crook et al., 2002). A second study, using also non-specific antibodies, found a significant increase in mGlu_2/3_R expression in the BA46 of schizophrenic subjects compared to controls, but not in other cortical regions including BA9 and BA11 (Gupta et al., 2005). The availability of specific antibodies allowed the evaluation of the protein expression of each subtype of Group II mGluRs. Corti et al. (2007) found a significant decrease in the dimeric form of mGlu_3_Rs in the PFC (BA10) of schizophrenic subjects compared to controls, with unaffected levels of the monomeric forms. Similarly, another study reported a decrease in mGlu_3_R protein in the PFC (BA46) of schizophrenic subjects, but not in other areas such as temporal or motor cortices (Ghose et al., 2009). This study reported unchanged mGlu_2_R protein expression in schizophrenia (Ghose et al., 2009), however, the antibody used to assess the mGlu_2_R immunoreactivity was not previously validated and was actually measuring the subtype 2 of the AMPA ionotropic glutamate receptor ^1^.

Four independent studies have investigated the radioligand binding density of mGlu_2/3_Rs in the postmortem brain of schizophrenic subjects. Gonzalez-Maeso et al. (2008), reported a decrease in the binding density of mGlu_2/3_Rs in the PFC (BA9) of schizophrenic subjects respect to matched controls using the mGlu_2/3_R antagonist [^3^H]LY341495. Other studies, however, reported no differences in mGlu_2/3_R binding density in the PFC (BA46) between schizophrenic subjects and controls when using either the mGlu_2/3_R agonist [^3^H]LY354740 (Frank et al., 2011) or the antagonist [^3^H]LY341495 (McOmish et al., 2016). Two studies have evaluated the mGlu_2/3_Rs density in other areas besides PFC, founding no differences between schizophrenia and control groups neither in the anterior cingulated cortex (BA24; Matosin et al., 2014; McOmish et al., 2016) nor in the visual cortex (BA17; McOmish et al., 2016).

Taking into account the different findings from postmortem studies the status of Group II mGluRs in schizophrenia remains unclear. Thus, further investigation of the level of expression and function of mGlu_2/3_Rs in postmortem human brain of schizophrenic subjects and controls is needed.

### Genetic Studies

While other factors besides genetics are definitely involved, investigation of the genetic alterations responsible for schizophrenia represents a useful approach to better understand the cause of the disease (Harrison and Weinberger, 2005). As mentioned above, mGlu_2_R gen (*GRM2*) has been mapped to chromosome 3p21.1–p21.2 (Marti et al., 2002a), and linkage studies of schizophrenia show no positive results regarding this region (Moreno et al., 2009). Moreover, in a population-based genetic study for candidate polymorphisms in alleles of the mGlu_2_R gene, no association was found between such polymorphisms and schizophrenia (Joo et al., 2001).

Genetic association analyses have consistently suggested an association between SNPs in the mGlu_3_R gene (*GRM3*) and schizophrenia (Fujii et al., 2003; Egan et al., 2004; Chen et al., 2005; Sartorius et al., 2008; Cherlyn et al., 2010) including a recent multi-stage schizophrenia genome-wide association study (Schizophrenia Working Group of the Psychiatric Genomics Consortium, 2014). However, this association was not replicated in other population-based genetic studies (Marti et al., 2002b; Tochigi et al., 2006). *GRM3* polymorphisms associated with schizophrenia are often located in a non-coding region. Therefore, the mechanism underlying the association between *GRM3* and schizophrenia is not clear. *GRM3* polymorphisms have been associated with negative symptom improvement during olanzapine treatment (Bishop et al., 2005). Egan et al. (2004) proposed a specific pathway by which *GRM3* genotype could alter the glutamatergic transmission leading to an increase in the risk for schizophrenia. These authors found an association between an intronic variation in *GRM3* and a reduced performance on cognitive tests of prefrontal and hippocampal function, which are schizophrenia related phenotypes. Moreover, in postmortem human PFC, *GRM3* variant carriers showed lower mRNA levels of the glial glutamate transporter EAAT2. Therefore, authors suggest that the pathophysiological mechanism underlying schizophrenia may involve altered mGlu3 transcription/expression and altered glutamate neurotransmission related to a reduced expression of the glial glutamate transporter EAAT2 (Egan et al., 2004).

## Group II mGluRs As Targets for Novel Antipsychotic Drugs

Metabotropic glutamate receptors have received significant interest as potential drug targets. Such interest is due to the belief that metabotropic receptor targeting provides a way for modulating glutamate tone and phasic release in a more subtle manner than that which can be achieved through glutamate ionotropic receptors. Specifically, emerging preclinical and clinical data suggest that activation of Group II mGluRs is a mechanistically novel and promising approach for the treatment of schizophrenia (Marek, 2004; Harrison, 2008; Krivoy et al., 2008; Sodhi et al., 2008; Conn et al., 2009; Chaki, 2010; Fell et al., 2012; Vinson and Conn, 2012; Wierońska et al., 2016).

### Preclinical Evidences of Antipsychotic Activity

Extensive preclinical data proved that orthosteric Group II mGluR agonists, including LY354740, LY379268, and LY404039 exhibit antipsychotic-like properties in animal models of schizophrenia. The dissociative drugs PCP and ketamine have been shown to increase the activity of glutamatergic synapses in the PFC (Adams and Moghaddam, 1998; Lorrain et al., 2003) and different studies have confirmed that Group II mGluR agonists are able to reverse this effect (Moghaddam and Adams, 1998; Marek et al., 2000; Lorrain et al., 2003). It has been also shown that the systemic administration of the mGlu_2/3_R agonists LY379268 and LY404039 is able to increase dopamine extracellular levels in rodent’s frontal cortex (Cartmell et al., 2001; Rorick-Kehn et al., 2007b). Increases in cortical dopamine levels have been linked to the improvement of negative symptoms in schizophrenia. Additionally, atypical antipsychotic drugs such as clozapine and risperidone also produce a cortical increase of this neurotransmitter (Cartmell et al., 2001). Besides dopamine, cortical serotonin is also enhanced by the systemic administration of mGlu_2/3_R agonists as well as by the atypical antipsychotic risperidone (Cartmell et al., 2001; Rorick-Kehn et al., 2007b). These neurochemical similarities between mGlu_2/3_R agonists and already known antipsychotic drugs provide support for the potential antipsychotic properties of the firsts.

In addition to this neurochemical evidence, Group II mGluR agonists have also shown the ability to reverse the behavioral effects induced by psychotomimetic drugs in several animal models predicting their potential as antipsychotic agents (Wierońska et al., 2016). In this sense, locomotor response to psychostimulants in rodents represents an animal correlate of schizophrenia positive symptoms (Arguello and Gogos, 2006). Regarding this aspect, the mGlu_2/3_R agonist LY354740 administered at a dose that did not affect spontaneous locomotor activity itself has proved to attenuate PCP-induced locomotor hyperactivity and stereotypies (Moghaddam and Adams, 1998). Similar results have been reported with other mGlu_2/3_R agonists, like LY404039 (Rorick-Kehn et al., 2007a,b), LY379268 (Cartmell et al., 2000), MGS0008 and MGS0028 (Nakazato et al., 2000). Moreover, the hyperactivity induced by amphetamine has also been shown to be inhibited by both LY379268 and LY404039 (Galici et al., 2005; Rorick-Kehn et al., 2007a,b). Furthermore, the agonists MGS0008, MGS0028, and LY404039 have been reported to inhibit conditioned avoidance responses (Takamori et al., 2003; Rorick-Kehn et al., 2007b) and LY354740 has also shown ability to prevent the PCP-induced deficits on a working memory task (Moghaddam and Adams, 1998), a paradigm that correlates with the cognitive dimension of schizophrenia symptoms (Arguello and Gogos, 2006). Interestingly, Group II mGluR agonists have also been shown to reverse the effects induced by 5-HT_2A_R hallucinogenic agonists. Thus, mGlu_2/3_R orthosteric agonists, such as LY379268 and LY354740, reduce the cellular (Zhai et al., 2003; Gonzalez-Maeso et al., 2008), electrophysiological (Marek et al., 2000) and behavioral (Gewirtz and Marek, 2000; Gonzalez-Maeso et al., 2008) effects induced by the hallucinogen 2,5-Dimethoxy-4-iodoamphetamine (DOI). Similar findings have been reported for the selective mGlu_2_R positive allosteric modulator (PAM) BINA (Benneyworth et al., 2007). In fact, in recent years, more attention has been paid to mGluRs PAMs, especially to those selective for mGlu_2_R subtype (Ellaithy et al., 2015). In this sense, several preclinical studies have shown efficacy for selective mGlu_2_R PAMs, i.e., CBiPES, JNJ-40411813, JNJ-42153605, and TASP0433864, in reversing psychotic-like symptoms (Johnson et al., 2005; Hiyoshi et al., 2014; Hikichi et al., 2015; Lavreysen et al., 2015). All of the above-mentioned findings support the potential use of mGlu_2/3_R agonists for the treatment of schizophrenia symptoms.

In regard to Group II mGluR agonists’ selectivity, Seeman et al. (2008) have suggested that the antipsychotic effects of these compounds may be due to their affinity for D2 receptors (Seeman et al., 2008). However, other laboratories have convincingly demonstrated that this direct effect of Group II mGluR agonists over dopamine receptors is not replicable (Fell et al., 2009; Zysk et al., 2011). In terms of subtype selectivity, it has been suggested that the antipsychotic effects exerted by Group II mGluR agonists are mediated by mGlu_2_R rather than by mGlu_3_R (Woolley et al., 2008; Fell et al., 2012; Vinson and Conn, 2012). This hypothesis is based on the results obtained in studies performed with mGlu_2_R-KO and mGlu_3_R-KO mice. Thus, antipsychotic actions of Group II agonists LY404039 and LY314582 (racemic mixture of LY354740) were absent in mGlu_2_R-KO mice but present in mGlu_3_R-KO mice, strongly implicating mGlu_2_R as the predominant player in this effect (Spooren et al., 2000; Fell et al., 2008). Similar results were observed for the Group II agonist LY379268 that reversed PCP- and amphetamine-evoked hyperactivity in wild type and mGlu_3_R-KO mice but not in mGlu_2_R-KO mice (Woolley et al., 2008). This finding is further supported by the results observed with selective mGlu_2_R PAMs mentioned above, which have shown efficacy in animal behavioral paradigms used to assess the antipsychotic activity regarding both positive-like symptoms and cognitive impairments (Johnson et al., 2003; Galici et al., 2005, 2006, Govek et al., 2005; Johnson et al., 2005; Pinkerton et al., 2005; Benneyworth et al., 2007; Duplantier et al., 2009; Hiyoshi et al., 2014; Hikichi et al., 2015; Lavreysen et al., 2015). The action of PAMs depends on the presence of a threshold level of agonist, since they do not activate the receptor directly. Hence, it has been postulated that PAMs may provide a safer and better tolerated therapeutic profile than orthosteric compounds, with a more regulated action and a lower potential receptor desensitization (Johnson et al., 2005; Urwyler, 2011). On the other hand, mGlu_3_Rs have been recently postulated as potential targets to treat the cognitive dysfunction in schizophrenia. Walker et al. (2015) showed that mGlu_3_Rs can influence synaptic plasticity within mice PFC and that the specific blockade of this receptor impairs learning in a mPFC-dependent fear extinction task. Thus, these authors propose selective PAMs of mGlu3Rs as a novel therapeutic strategy for enhancing prefrontal function in schizophrenic patients (Walker et al., 2015).

### Clinical Evidences of Antipsychotic Activity

The selective Group II mGluR agonists have been well-characterized and optimized and have entered into clinical trials for treatment of schizophrenia. The oral prodrug of LY404039, (1R,4S,5S,6S)-2-thiabicyclo[3.1.0]- hexane-4,6-dicarboxylic acid,4-[(2S)-2-amino-4-(methylthio)-1-oxobutyl]amino-, 2,2-dioxide monohydrate (LY2140023) developed by Eli Lilly and Co, showed significant antipsychotic efficacy for both positive and negative symptoms with no major side effects in a trial involving patients suffering from schizophrenia and also showed a better metabolic profile than the comparator olanzapine (Patil et al., 2007). Unfortunately, in the follow-up study, neither LY2140023 nor the comparator olanzapine were more efficacious than placebo as measured by the Positive and Negative Syndrome Scale (PANSS) total score due to higher-than expected placebo response (Kinon et al., 2011), thus, the results of this study were considered to be inconclusive (Kinon et al., 2011). In a more recent multicenter, randomized, double-blind, phase II study, LY2140023 monohydrate was again tested against placebo and the active control risperidone in schizophrenic patients with an acute exacerbation of symptoms. The primary outcome assessed change from baseline in the PANSS total score in an overall schizophrenia population and a predefined subpopulation which excluded non-Hispanic white patients with the A/A genotype at the serotonin 2A receptor (5-HT_2A_R) single nucleotide polymorphism rs7330461 (Downing et al., 2014). Neither LY2140023 dose showed significant improvement compared to placebo in either population. Conversely, risperidone showed a better efficacy than placebo in both populations (Downing et al., 2014). Finally, another study found no benefit of adjunctive treatment with LY2140023 versus placebo for negative symptoms in patients with schizophrenia receiving treatment with second-generation antipsychotics (Stauffer et al., 2013). The mGlu_2_R PAM JNJ-40411813/ADX71149 from Janssen Pharmaceuticals, Inc. and Addex Therapeutics has also been evaluated in clinical trials for schizophrenia treatment. Data reported in 2012 showed that JNJ-40411813/ADX71149 met the primary objectives of safety and tolerability and demonstrated an effect in patients with residual negative symptoms (Hopkins, 2013). The latest data in two phase-1 studies showed efficacy of the drug reducing the continuity of attention score, improving the quality of episodic memory and reducing the ketamine-induced negative symptoms in healthy volunteers (Salih et al., 2015). Another mGlu_2_R PAM that has advanced into clinical trials is the AZD8529 from AstraZeneca. Despite this compound failed to be effective in a phase 2 study when administered as monotherapy at a single dose in schizophrenic patients, it remains to be determined whether different treatment regimens or adjunct treatment would provide benefit (Litman et al., 2014).

## The Role of the 5-Ht_2A_R/mGlu_2_R Heterocomplex

5-HT_2A_R and mGlu_2_R have been both implicated in the pathophysiology of schizophrenia and also have been considered as targets for antipsychotic drug development. Previous electrophysiological (Marek et al., 2000), cellular (Benneyworth et al., 2007), neurochemical (Martin-Ruiz et al., 2001), and behavioral (Gewirtz and Marek, 2000) data have suggested an interaction between 5-HT_2A_ and mGlu_2_ receptors. At present, it has been convincingly proved the existence of a specific functional heteromeric complex formed by 5-HT_2A_ and mGlu_2_ receptors through which serotonin and glutamate ligands modulate the pattern of G protein-coupling in living cells (Gonzalez-Maeso et al., 2008; Moreno et al., 2012; Baki et al., 2016).

**FIGURE 2:**
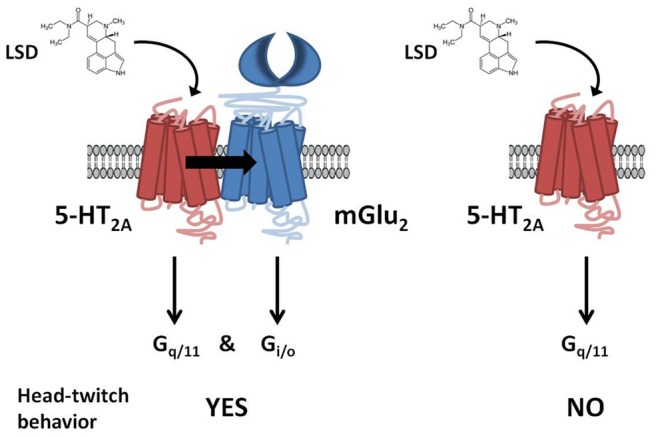
**G protein-dependent signaling and behavioral responses that require the 5-HT_2A_R/mGlu_2_R heterocomplex.** LSD acting at the 5-HT_2A_R/mGlu_2_R heterocomplex activates both G_q/11_- and G_i/o_-dependent signaling. In contrast, when 5-HT_2A_R and mGlu_2_R are prevented from forming a receptor heterocomplex, activation of 5-HT_2A_R by LSD elicits characteristic signaling of G_q/11_-protein subtypes. Head-twitch behavior is reliably and robustly elicited by hallucinogenic 5-HT_2A_R agonists, and is absent in mGlu_2_R-KO mice. Adapted from Gonzalez-Maeso (2011).

This serotonin-glutamate heterocomplex has been involved in the mechanism of action of both hallucinogenic (Gonzalez-Maeso et al., 2003, 2007; Moreno et al., 2011a) and antipsychotic drugs (Fribourg et al., 2011). Thus, it has been reported that mGlu_2_R is necessary for at least some of the cellular and behavioral responses induced by hallucinogenic 5-HT_2A_R agonists such as lysergic acid diethylamide (LSD). It has been shown in [^35^S]GTPγS binding assays followed by immunoprecipitation with anti-G_q/11_ or anti-G_i1,2,3_ antibodies that the hallucinogenic 5-HT_2A_R agonist DOI activates both G_q/11_ and G_i_ proteins only when the 5-HT_2A_R is expressed as a receptor heterocomplex with the mGlu_2_R (Gonzalez-Maeso et al., 2008). Moreover, the head-twitch response was not produced by the hallucinogens DOI and LSD in mGlu_2_R-KO mice (Moreno et al., 2011a). Furthermore, it has been recently proved that the disruption of heteromeric expression with mGlu_2_R attenuates the psychosis-like effects induced in mice by hallucinogenic 5-HT_2A_R agonists (Moreno et al., 2012; **Figure 2**). These authors, not only validate the 5-HT_2A_/mGlu_2_ receptor heterocomplex as necessary for the behavioral effects induced by LSD-like drugs in rodents, but also provide the first evidence for the specific residues responsible for a G protein-coupled receptor (GPCR) heteromeric complex formation (Moreno et al., 2012).

Atypical antipsychotic drugs, such as clozapine and risperidone, have a high affinity for the serotonin 5-HT_2A_R, which preferred signaling pathway is via G_q/11_ proteins. Closely related non-antipsychotic drugs, such as ritanserin and methysergide, also block 5-HT_2A_R function, but they lack comparable neuropsychological effects. In this regard, it has been reported that these ligands inputs are actually integrated by the 5-HT_2A_R/mGlu_2_R heterocomplex that modulates signaling outputs and behavioral changes (Fribourg et al., 2011). Thus, serotonergic and glutamatergic drugs would bind to the 5-HT_2A_R/mGlu_2_R heterocomplex, which then balances G_i/o_- and G_q/11_-dependent signaling. The authors also state that 5-HT_2A_R/mGlu_2_R -mediated changes in G_i/o_ and G_q/11_ activity could predict the psychoactive behavioral effects of different pharmacological compounds.

Importantly, it has been demonstrated a dysregulation in the binding density of the receptors comprising this heterocomplex in postmortem PFC of schizophrenic subjects (Gonzalez-Maeso et al., 2008). Thus, increased 5-HT_2A_R and decreased mGlu_2/3_R binding was found in schizophrenic subjects compared to matched controls (Gonzalez-Maeso et al., 2008). Interestingly, 5-HT_2A_R density was comparable to control values in those subjects that were under antipsychotic treatment at time of death, whereas mGlu_2/3_R density remains decreased. Furthermore, the ligand binding interaction between the components of the 5-HT_2A_/mGlu_2_ receptor heterocomplex was found up-regulated in the postmortem PFC of schizophrenic subjects as compared with controls (Moreno et al., 2012). Additionally, at the level of signaling, a recent study by same authors showed that mGlu_2_R-dependent activation of G_q/11_, but not G_i/o_ proteins, is reduced in the postmortem PFC from schizophrenic patients (Moreno et al., 2016). Moreover, recent studies have reported altered densities and behavioral functions of 5-HT_2A_ and mGlu_2_ receptors in different animal models that resemble some aspects of schizophrenia. Thus, in frontal cortex of mice born to influenza virus-infected mothers, stressed mothers or lipopolysaccharide (LPS)-treated mothers the 5-HT_2A_R is upregulated (Moreno et al., 2011b; Holloway et al., 2013; Wischhof et al., 2015) and the mGlu_2_R receptor is downregulated (Moreno et al., 2011b; Holloway et al., 2013). Furthermore, these changes are translated into behavioral alterations, since increased head-twitch response to the hallucinogenic 5-HT_2A_R agonist DOI and decreased mGlu_2_-dependent antipsychotic-like effect of the mGlu_2/3_ agonist LY379268 were observed in these three studies (Moreno et al., 2011b; Holloway et al., 2013; Wischhof et al., 2015).

A pharmacogenetic analysis of the efficacy of LY2140023 monohydrate in the treatment of schizophrenia has demonstrated a genetic association between several single nucleotide polymorphisms located in the gene encoding the 5-HT_2A_R and the response to LY2140023 treatment (Liu et al., 2012). Thus, a 30-point PANSS total reduction was seen in schizophrenic patients in the most responsive genotype group that presented the single nucleotide polymorphism rs7330461 for the 5-HT_2A_R gene (Liu et al., 2012). Additionally, a recent study has confirmed that the T/T genotype at rs7330461 is consistently associated with an increased treatment response to pomaglumetad methionil (LY2140023) compared to the A/A genotype (Nisenbaum et al., 2016).

All these facts point to a putative role of the 5-HT_2A_R/mGlu_2_R heterocomplex in the antipsychotic-like properties of the Group II mGluR agonists that could also explain the controversial results reported in clinical trials. In this way, it has been demonstrated that chronic atypical antipsychotics downregulate the transcription of mGlu_2_R through epigenetic modifications (Kurita et al., 2012). This change occurs in concert with a 5-HT_2A_R-dependent up-regulation and increased binding of histone deacetylase 2 to the *mGlu2* promoter (Kurita et al., 2012). This decrease in the mGlu_2_R expression could induce a lower response to Group II mGluR agonists as LY2140023 in patients previously treated with atypical antipsychotics. Accordingly, a recent study has reanalyzed previous clinical data on LY2140023 treatment defining two patients subpopulations based upon medication exposure during the 2 years before study entry (Kinon et al., 2015). This analysis has demonstrated that patients previously treated with antipsychotics with prominent dopamine 2 receptor antagonist activity who were subsequently treated with LY2140023 monohydrate showed a significantly greater improvement on the PANSS total score from baseline than placebo treated patients. Conversely, patients previously treated with antipsychotics with prominent 5-HT_2A_R antagonist activity demonstrated no greater response than placebo (Kinon et al., 2015). Thus, as LY2140023 monohydrate treatment is targeted to mGlu_2_R receptor activation it will induce lower efficacy if the mGlu_2_R receptor levels are reduced as a consequence of previous treatment with atypical antipsychotics.

## Conclusion and Future Directions

Several studies have showed that Group II mGluR agonists exhibit antipsychotic-like properties in preclinical assays. However, when these compounds have been used in human clinical trials the results have been controversial. Recent data suggest that this apparent lack of efficacy in schizophrenic patients may be related to previous exposure to atypical antipsychotics. Moreover, pharmacogenetic assays have demonstrated the influence of genetic variants on response to Group II mGluR agonists in patients with schizophrenia. The fact that Group II mGluRs represent a new target for the treatment of schizophrenia supports the need for additional investigation to establish the real efficacy of these new compounds. Moreover, it is mandatory to clarify if specific subgroups of patients could obtain a greater benefit from using these new drugs.

## Author Contributions

All authors listed, have made substantial, direct and intellectual contribution to the work, and approved it for publication.

## Conflict of Interest Statement

The authors declare that the research was conducted in the absence of any commercial or financial relationships that could be construed as a potential conflict of interest.

## Footnotes

1 http://www.abcam.com/Ionotropic-Glutamate-receptor-2-antibody-ab52176.html
